# Transition from Anomalous Hall Effect to Topological Hall Effect in Hexagonal Non-Collinear Magnet Mn_3_Ga

**DOI:** 10.1038/s41598-017-00621-x

**Published:** 2017-03-31

**Authors:** Z. H. Liu, Y. J. Zhang, G. D. Liu, B. Ding, E. K. Liu, Hasnain Mehdi Jafri, Z. P. Hou, W. H. Wang, X. Q. Ma, G. H. Wu

**Affiliations:** 1 Department of Physics, University of Science and Technology Beijing, Beijing, 100083 People’s Republic of China; 2 School of Material Science and Engineering, Hebei University of Technology, Tianjin, 300130 People’s Republic of China; 3 Beijing National Laboratory for Condensed Matter Physics, Institute of Physics, Chinese Academy of Sciences, Beijing, 100190 People’s Republic of China

## Abstract

We report experimental observation of large anomalous Hall effect exhibited in non-collinear triangular antiferromagnet D0_19_-type Mn_3_Ga with coplanar spin structure at temperatures higher than 100 K. The value of anomalous Hall resistivity increases with increasing temperature, which reaches 1.25 μΩ · cm at a low field of ~300 Oe at room temperature. The corresponding room-temperature anomalous Hall conductivity is about 17 (Ω · cm)^−1^. Most interestingly, as temperature falls below 100 K, a temperature-independent topological-like Hall effect was observed. The maximum peak value of topological Hall resistivity is about 0.255 μΩ · cm. The appearance of the topological Hall effect is attributed to the change of spin texture as a result of weak structural distortion from hexagonal to orthorhombic symmetry in Mn_3_Ga. Present study suggests that Mn_3_Ga shows promising possibility to be antiferromagnetic spintronics or topological Hall effect-based data storage devices.

## Introduction

**Figure 1:**
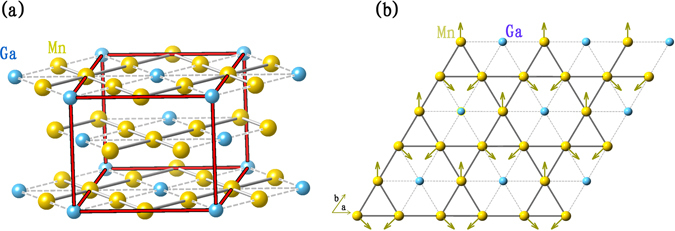
(**a**) The hexagonal structure of Mn_3_Ga, (**b**) An individual *a*-*b* plane of Mn_3_Ga. Mn moments (arrows) form a non-collinear AFM configuration^6, 7^.

Mn_3_Ga compound has attracted much attention in recent years because its multiple phases exhibit interesting structural, magnetic and electron-transport properties, potentially useful in spintronic and magnetic applications^1^. It has been reported to have tetragonal, hexagonal and face-centered cubic crystal structures when subjected to different heat treatments^2–4^. Hexagonal Mn_3_Ga (70–74 at.% Mn) has a D0_19_-type structure with space group P63/mmc^5^, as shown in Fig. 1(a). All Mn moments lie in the *a-b* plane and form a triangular antiferromagnetic spin structure (see Fig. 1(b)), with each layer of Mn triangles stacked along the *c* axis^6^. In each layer, Mn atoms form a Kagome lattice, with Ga sitting at the center of a hexagon. This hexagonal structure is equivalent to an orthorhombic structure, denoted by red line in Fig. 1(a). Neutron diffraction measurements confirmed that directions of magnetic moments in the same layer are not equally separated by 120°, therefore they do not fully cancel each other, resulting in a weak ferromagnetic phase with a small formula moment^6^. Decreasing temperature below 170 K, hexagonal structure slightly distorts to an orthorhombic one, giving rise to a large deviation of spin structure from ideal triangular spin configuration compared with the hexagonal structure^7^.

Anomalous Hall effect (AHE) in non-collinear antiferromagnets have attracted much attention recently due to its potential applications in spintronic devices, being proposed to be used for spin-transfer torque and spin pumping^8^. In addition, no stray field and reduction of switching current are among the features making antiferromagnets attractive compared to conventionally used ferromagnets in design of spintronic devices. Mn_3_Sn and Mn_3_Ge have same hexagonal structure as Mn_3_Ga^9^. It has been predicted theoretically by first-principle calculations in 2014 that non-collinear antiferromagets Mn_3_Sn and Mn_3_Ge exhibit large AHE due to non-vanishing Berry curvature^9^. One year later, the large AHE at room temperature was discovered experimentally in both alloys^10, 11^. It is generally considered that an ordinary antiferromagnet with collinear moments should not exhibit AHE due to disappearance of Berry phase. However, there is a non-vanishing Berry phase in non-collinear spin arrangment antiferromagnets, which leads to an anomalous Hall conductivity^12, 13^. Furthermore, it has been suggested that fictitious magnetic field caused by Berry phase may induce a nontrivial Hall effect termed as topological Hall effect (THE)^14^. THE has been proposed to appear in presence of non-coplanar spin configurations and therefore it may occur in antiferromagnetic materials with highly non-collinear and non-coplanar spin structure^15^. Onoda *et al*. pointed out that there are two mechanisms causing THE. One is the presence of inequivalent multiple loops in the unit cell, i.e. THE in Nd_2_Mo_2_O_7_^16^, while other one is spin texture hosting the spin chirality, whose size is much larger than the lattice constant, i.e. THE in MnGe^17^, FeGe^18^, MnSi^19^ with B20 crystal structure and MnNiGa with a layered Ni_2_In-type hexagonal structure^20^.

In this study, we report that besides the large AHE in non-collinear antiferromagnet Mn_3_Ga at room temperature, like in Mn_3_Sn and Mn_3_Ge^10, 11^, a large topological-like Hall effect appears at temperatures below 100 K accompanying with a weak structural transition. It is analyzed that THE is attributed to non-coplanar spin systems due to a small component of spin cant towards *c* axis in low temperature phase.

## Results and Discussion

**Figure 2:**
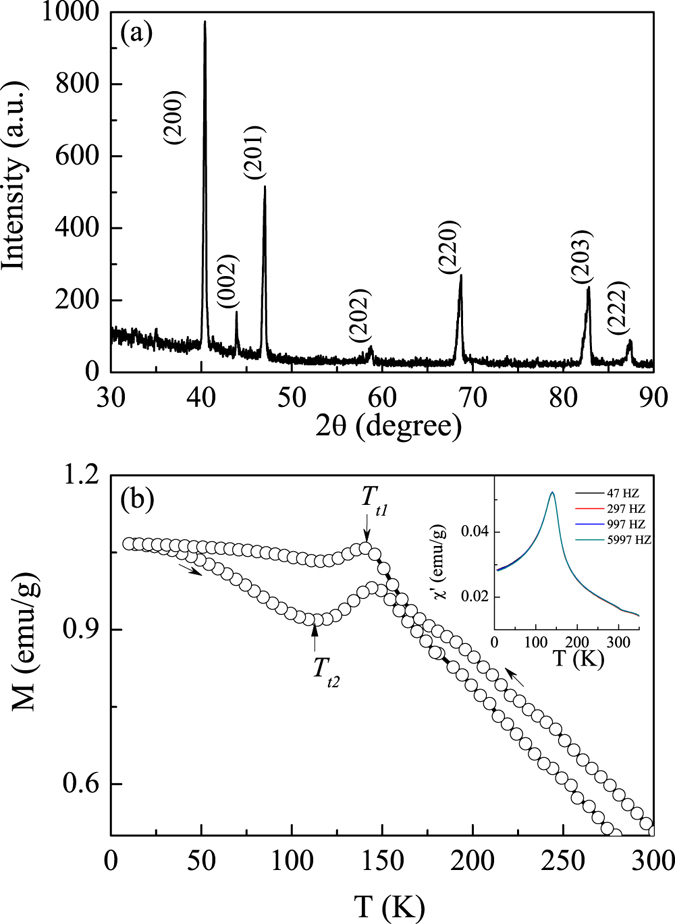
(**a**) XRD patterns measured at room temperature for Mn_3_Ga plate sample. (**b**) FC and FH curves measured at 100 Oe field for Mn_3_Ga. Inset of Fig. 2(b) is temperature dependence of the real part (*χ*′) of AC susceptibility measured at different frequencies with an ac magnetic field of 5 Oe after zero field cooling to 5 K.

Figure 2(a) shows XRD patterns measured at room temperature for Mn_3_Ga plate sample heat treated at 893 K. All diffraction peaks are indexed to be D0_19_ type hexagonal structure with space group P63/mmc. The lattice parameters were calculated to be *a* = 5.4010 Å and *c* = 4.3945 Å, which are close to those reported previously^7^.

Figure 2(b) shows field cooling (FC) and field heating (FH) curves measured at 100 Oe field from 350 K → 5 K → 350 K for hexagonal Mn_3_Ga. The magnetization gradually increases with decreasing temperature up to *T*_*t1*_ = 140 K, then followed by a great decrease with further cooling within 140~111 K. During heating, a reverse jump starting at *T*_*t2*_ = 100 K and finishing at 144 K can be clearly observed. The jump of M-T curve and existence of hysteresis between FC and FH jumps, together with the previous report that XRD confirmed hexagonal structure slightly distorts to an orthorhombic one at temperatures lower than 170 K in this alloy^7^, it is implied that a weak structural transition occurs in our alloy. AC susceptibility varying with temperature under different driving frequencies in the presence of 5 Oe ac field after zero field cooling to 5 K was measured. Inset of Fig. 2(b) shows the temperatre dependence of real part (*χ*′) of AC susceptibility. *χ*′-T curve shows one clear peak at 140 K, which does not present any frequency dispersion, meet with the feature of the structural transition^21^.

**Figure 3:**
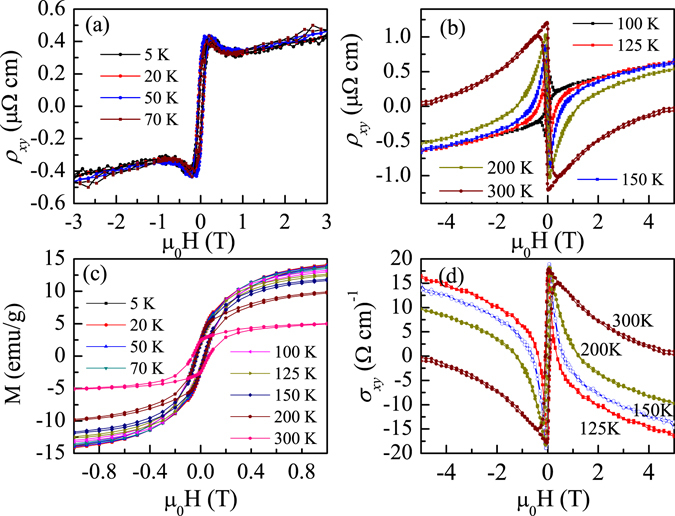
(**a** and **b**) show Hall resistivity *ρ*_*xy*_ as a function of magnetic field at different temperatures, (**c**) is magnetic hysteresis curves at different temperatures, (**d**) shows magnetic field dependence of Hall conductivity σ_xy_.

Figure 3(a) and (b) show Hall resistivity *ρ*_*xy*_ as a function of magnetic field at different temperatures. *ρ*_*xy*_ increases rapidly with increasing magnetic field in very low fields exhibiting a clear hysteresis loop. Notably, at temperatures below 100 K, the shape and magnitude of *ρ*_*xy*_ almost does not change with temperature, as shown in Fig. 3(a). *ρ*_*xy*_ is negative for negative fields and positive for positive fields. At temperatures higher than 100 K, all the curves have similar shape. *ρ*_*xy*_ increases rapidly initially followed by a great decrease with increasing field, peak value of *ρ*_*xy*_ increases with increasing temperature, while at higher fields *ρ*_*xy*_ decreases with increasing temperature. Furthermore, the sign of spontaneous Hall effect changes at temperatures higher than 100 K(see Fig. 3(b)). This temperature range for sign change is consistent with that of structural transition from hexagonal to orthorhombic phase. It was observed that one half of the curve of *ρ*_*xy*_ at 100 K has the characteristic of lower temperature curves and another half has shape of high temperature curves, implying that the sample stays an intermediate transition state at 100 K.

Figure 3(c) shows magnetic hysteresis curves (M-H) at different temperatures, exhibiting the same hysteresis behavior as Hall resistivity but with different curve shape, which is a striking feature for Hall effect of Mn_3_Ga. The magnitude of magnetization decreases with increasing temperature. The saturation magnetization decreases rapidly from 12 to 5 emu/g with temperature increasing from 125 K to 300 K. However, the peak value of *ρ*_*xy*_ increases with decreasing saturation magnetization, which reaches 1.25 μΩ cm at a small field of ~300 Oe at room temperature. Since the shape of magnetization curves is different from that of Hall resistivity in this temperature range, *ρ*_*xy*_ cannot be attributed to common AHE as observed in ferromagnets. Simultaneously, previous neutron diffraction measurements and theoretical analysis clarified inverse triangular spin structure of hexagonal Mn_3_Ga alloy with Mn moments lying in *a*-*b* plane^6^. Because of the in-plane coplanar magnetic spin structure, the shape deviation from the magnetization curves cannot attribute to THE, which stems from non-coplanar spin structure. Behavior of these curves for Mn_3_Ga is similar to that observed in Mn_3_Sn alloy^10^, which is considered to be arising from non-collinear antiferromagnetic spin structure. This additional contribution to the AHE, associated with non-vanishing Berry curvature due to non-collinear spin structure, results in the Hall resistivity features that do not resemble the magnetization curves. The value of anomalous Hall resistivity at room temperature is about 0.5~4 μΩ cm in Mn_3_Sn and Mn_3_Ge single crystal samples^10, 11^, which is different in different crystallographic directions. Furthermore, the AHE present opposite sign in specified crystallographic directions (see Fig. 2 in both refs 10 and 11), showing strong anisotropic behavior. Our Mn_3_Ga sample is polycrystalline, the anomalous Hall resistivity is an average effect, thus the shape of curves is a little different from the single crystals of Mn_3_Sn and Mn_3_Ge.

Comparably, as temperature falls below 100 K, although magnetization decreases with increasing temperature, *ρ*_*xy*_-H curves almost do not change with temperature. Simultaneously, a hump-like anomaly can be clearly observed, which is considered as a unique symbol of THE^17^. These feathers indicate the appearance of THE in our Mn_3_Ga, as has been reported in MnSi^17^, FeGe^18^, MnGe^19^, and MnNiGa^20^ alloys, which has been a distinction for the prominent non-planar magnetic configurations, i.e. spin chirality or magnetic winding. Hexagonal structure of Mn_3_Ga can slightly distort to an orthorhombic one at low temperatures, giving rise to a larger deviation of spin structure from the ideal triangular spin configuration compared with hexagonal structure^7^. This may result in a small component of spins canting towards the *c* axis at low temperature phase, as evidenced by increase of magnetization. This is necessary for the formation of scalar spin chirality. Thus, an interesting THE is observed.

The anomalous Hall conductivity (AHC) *σ*_*xy*_ = −*ρ*_*xy*_/*ρ*_*xx*_^2^ (satisfy *ρ*_*xx*_ ≫ *ρ*_*xy*_), where *ρ*_*xx*_ is normal resistivity, has been calculated when temperature is higher than 100 K, as shown in Fig. 3(d). *σ*_*xy*_ increases firstly, reaching a large value of ~17 (Ω · cm)^−1^ at a small field of ~300 Oe, and then followed by a great decrease. The absolute value of *σ*_*xy*_ at 5 T increases with decreasing temperature, which is only 0.6 (Ω · cm)^−1^ at 300 K and nearly 15 (Ω · cm)^−1^ at 125 K. Theoretical calculations predicted that non-collinear antiferromagnet Mn_3_Ga exhibits the smallest AHC among Mn_3_Ge, Mn_3_Sn, and Mn_3_Ga^22^. Previous reports found that the AHC for Mn_3_Ge and Mn_3_Sn are 50, 20 (Ω · cm)^−1^, respectively^10, 11^. Both values are larger than the AHC for Mn_3_Ga, which has a value of 17 (Ω · cm)^−1^. These experimental results are fully consistent with recent theoretical predication^22^.

**Figure 4:**
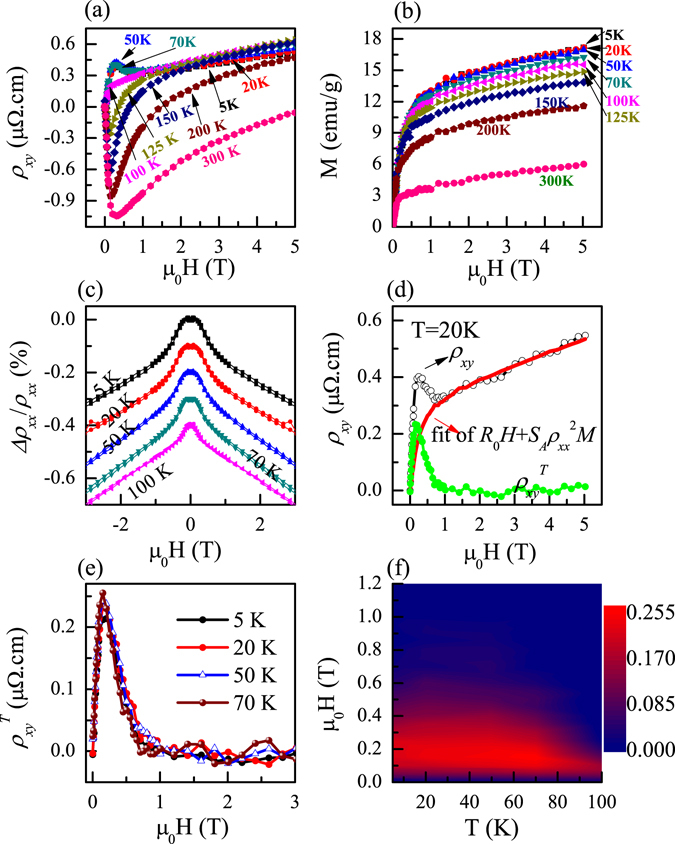
(**a**) Initial Hall resistivity *ρ*_*xy*_ measured at various temperatures from 5 K to 300 K; (**b**) M-H curves measured for Mn_3_Ga thin plate with field direction perpendicular to the sample plane; (**c**) Magnetoresistivity measured at different temperatures; (**d**) The representative *ρ*_*xy*_ *-*H curve (black circle) measured at 20 K, calculated ${R}_{0}H+{S}_{A}{\rho }_{xx}^{2}M$ curve (red line), and derived topological resistivity $({\rho }_{xy}^{T})$; (**e**) ${\rho }_{xy}^{T}$ at various temperatures extracted from *ρ*_*xy*_ *-*T curves in (**a**); (**f**) The contour mapping of extracted ${\rho }_{xy}^{T}$ as a function of the external magnetic field H and temperature.

To investigate low temperature THE deeply, the initial Hall resistivity *ρ*_*xy*_ of Mn_3_Ga was measured at different temperatures, as shown in Fig. 4(a). In this situation, total Hall resistivity *ρ*_*xy*_ is contributed by H-linear normal Hall resistivity ${\rho }_{xy}^{N}$, abnormal Hall resistivity ${\rho }_{xy}^{A}$, and the topological Hall resistivity ${\rho }_{xy}^{T}$, therefore ${\rho }_{xy}={\rho }_{xy}^{N}+{\rho }_{xy}^{A}+{\rho }_{xy}^{T}={R}_{0}H+{S}_{A}{\rho }_{xx}^{2}M+{\rho }_{xy}^{T}$, where *R*_*0*_ is ordinary Hall coefficient, ${S}_{A}{\rho }_{xx}^{2}$ corresponds to AHE coefficient due to magnetization behavior and *S*_*A*_ is H-independent parameter, H is magnetic field perpendicular to the sample plane, and *M* is the corresponding magnetization. To calculate ${\rho }_{xy}^{T}$, we must subtract both normal and anomalous Hall resistivity from total Hall resistivity *ρ*_*xy*_. It should be pointed out that these equations are valid in weak magnetic field for which *ω*_c_ *τ* ≪ 1, where *ω*_c_ = *eB*/*m* is the cyclotron frequency, *τ* = *m*/*ne* ^2^ *ρ*_*xx*_ is electron scattering time and *m* is electron mass^15^. Normal Hall coefficient *R*_*0*_, effective carrier density *n*, *τ* and calculated *ω*_*c*_ *τ* up to the maximum applied field of 5 T at different temperatures are listed in Table 1. In the present study, we obtained *ω*_c_ *τ* = *BR*_*0*_/*ρ*_*xx*_ ~ 0.001, which satisfies the condition of *ω*_c_ *τ* ≪ 1^15^. This indicates the feasibility of above method to obtain topological resistivity.

**Table 1:** Normal Hall coefficient (*R*_*0*_), effective carrier density (*n*), electron scattering time (*τ*), calculated *ω*_*c*_ *τ* (*ω*_c_ the cyclotron frequency), *S*_*A*_ corresponds to AHE coefficient due to magnetization behavior, and the maximum value of ${{\boldsymbol{\rho }}}_{{\boldsymbol{xy}}}^{{\boldsymbol{T}}}$ at different temperatures.

T (K)	*R*_*0*_ (Ωm/T)	*n* (holes/m^3^)	*τ* (*s*)	*ω*_*c*_ *τ*	*S*_*A*_	ρ_xy_^T^ peak (μΩ cm)
5	2.65 × 10^−10^	2.36 × 10^28^	1.08 × 10^−15^	0.000945	0.01593	0.214
20	3.06 × 10^−10^	2.04 × 10^28^	1.23 × 10^−15^	0.00108	0.01547	0.234
50	3.48 × 10^−10^	1.79 × 10^28^	1.30 × 10^−15^	0.00114	0.01415	0.242
70	4.33 × 10^−10^	1.45 × 10^28^	1.50 × 10^−15^	0.00132	0.01219	0.255

*τ*, *ω*_c_, *ω*_*c*_ *τ* are calculated for the maximum applied field of 5 T.

Figure 4(b) shows magnetization curves at different temperatures with field perpendicular to the sample plane. Figure 4(c) shows the perpendicular magnetoresistance Δ*ρ*_*xx*_/*ρ*_*xx*_. The topological Hall resistivity vanishes when ferromagnetic spin state is induced as *H* > *H*_*c*_, where *H*_*c*_ is the critical field inducing transition from non-collinear antiferromagnetic to the ferromagnetic collinear state.

Therefore, when magnetic field *H* is higher than *H*_*c*_, only ordinary Hall effect and AHE exist in the sample, namely ${\rho }_{xy}={\rho }_{xy}^{N}+{\rho }_{xy}^{A}={R}_{0}H+{S}_{A}{\rho }_{xx}^{2}M$. It can be derived that $\frac{{\rho }_{xy}}{H}$ has a linear relationship with $\frac{{\rho }_{xx}^{2}M}{H}$ as *H* > *H*_*c*_. Therefore coefficient *R*_0_ and *S*_*A*_ can be determined from the intercept and the slope of the linear fitting of $\frac{{\rho }_{xy}}{H}$ versus $\frac{{\rho }_{xx}^{2}M}{H}$ in high magnetic field regions. Table 1 gives *R*_*0*_ and *S*_*A*_ at different temperatures. It is observed that *R*_0_ is positive, ~10^−10^ Ωm/T, suggesting hole-like conduction of the sample. The corresponding effective charge carrier density n is ~10^28^ holes/m^3^. Simultaneously, *R*_*0*_ increases with increasing temperature from 5 K to 70 K. The difference between total Hall resistivity ${\rho }_{xy}$ and fitting curve ${R}_{0}H+{S}_{A}{\rho }_{xx}^{2}M$ at *H* < *H*_*c*_ equals to topological resistivity ${\rho }_{xy}^{T}$. For instance, in Fig. 4(d), at *T* = 20 K, calculated ${R}_{0}H+{S}_{A}{\rho }_{xx}^{2}M$ curve (red line) using above fitted *R*_*0*_ and *S*_*A*_ values, and derived ${\rho }_{xy}^{T}$ curve (green line) from the analysis are shown. At *H* > *H*_*c*_, it is observed that value of ${R}_{0}H+{S}_{A}{\rho }_{xx}^{2}M$ is in good agreement with experimental data.

Figure 4(e) shows ${\rho }_{xy}^{T}$ at different temperatures extracted from total Hall resistivity *ρ*_*xy*_. It was found that topological Hall resistivity is nearly temperature independent, which is in agreement with the feature of THE. Furthermore, the value of topological Hall resistivity increases initially and then decreases with further increasing field, showing a peak behavior. This hump-like anomaly is considered as a typical symbol of THE^19, 23^. The peak value of ${\rho }_{xy}^{T}$ for each temperature increases rather slowly with increasing temperature, as given in Table 1. Maximum peak value of ${\rho }_{xy}^{T}$ is about 0.255 μΩ · cm, which is larger than that for bulk MnNiGa (${\rho }_{xy}^{T}$ = 0.15 μΩ · cm)^20^ and MnGe (${\rho }_{xy}^{T}$ = 0.16 μΩ · cm)^17^. Contour mapping of derived ${\rho }_{xy}^{T}$ value is shown in Fig. 4(f). The magnitude of ${\rho }_{xy}^{T}$ is nearly T-independent across a broad temperature range from 90 K to 5 K. It has been suggested that the THE is induced by fictitious magnetic field caused by non-vanishing Berry phase in non-collinear spin arrangment antiferromagnet^12^. THE is generally considered as a hallmark of magnetic skyrmions that has topological spin textures, as observed in MnGe^17^, MnSi^19^ and MnNiGa^20^ alloys, so we can speculate that our sample may possess magnetic skyrmions, which are promising materials for technological applications in magnetic storage and other spintronic applications^24^. It would be amusing to confirm this possibility in future studies.

## Conclusion

In summary, we report an experimental observation that a large anomalous Hall effect exhibits in non-collinear triangular antiferromagnet Mn_3_Ga at temperatures above 100 K. The value of anomalous Hall resistivity increases with increasing temperature. As the temperature is lower than 100 K, a topological-like Hall effect up to 0.255 μΩ · cm is observed due to the spin texture change resulting from the weak structural distortion from hexagonal to orthorhombic phase in Mn_3_Ga. The present study provides a possible candidate material for magnetic skyrmions, which will have great potential applications in future high-performance spintronic devices.

## Methods

### Sample preparation and structure characterization

Polycrystalline Mn_3_Ga button ingot was prepared using an arc melting furnace in argon atmosphere from high purity (99.99 %) elemental metals. The ingot (~5 g) was heat-treated at 893 K for 3 days in vacuum followed by quenching into ice water. The structure of the sample was characterized by X-ray diffraction (XRD) technique using a Philips X’Pert MPD instrument with Cu Kα radiation.

### Magnetic and transport measurements

To measure magneto-transport properties, the polycrystalline crystal was milled into a bar-shape with a typical size of about 3.0 × 1.0 × 0.04 mm^3^. The longitudinal and Hall resistivities were measured using a standard four probe method on the same sample with Physical Properties Measurement System (PPMS, Quantum Design, Inc.). Field dependence of Hall resistivity was obtained by subtracting the longitudinal resistivity component, while the field dependence of the longitudinal resistivity was obtained by subtracting the Hall resistivity component. The zero-field remanent magnetization, *M*, was also measured using same field-cooling procedures as used in both longitudinal and Hall resistivity measurements using PPMS. Magnetization hysteresis curves and AC susceptibility were also measured for the same bar sample with PPMS system.
